# Serum Zonulin and Endotoxin Levels in Exceptional Longevity versus Precocious Myocardial Infarction

**DOI:** 10.14336/AD.2017.0630

**Published:** 2018-04-01

**Authors:** Pedro Carrera-Bastos, Óscar Picazo, Maelán Fontes-Villalba, Helios Pareja-Galeano, Staffan Lindeberg, Manuel Martínez-Selles, Alejandro Lucia, Enzo Emanuele

**Affiliations:** ^1^Clinical Research Center, Faculty of Medicine, Center for Primary Health Care Research, Lund University, Malmö, Sweden.; ^2^NutriScience - Education and Consulting, Lisbon, Portugal.; ^3^European University of Madrid, Spain.; ^4^Research Institute of Hospital 12 de Octubre (’i+12’), Madrid, Spain.; ^5^Servicio de Cardiología, Hospital Universitario Gregorio Marañón, CIBERCV, Universidad Europea, Universidad Complutense, Madrid, Spain; ^6^2E Science, Robbio, Pavia, Italy.

**Keywords:** centenarians, longevity, inflammation, endotoxemia

## Abstract

Endotoxemia-induced inflammation has been associated with insulin resistance and atherosclerosis, ultimately increasing the risk of coronary heart disease. Increased intestinal permeability is an important event leading to endotoxemia. This study aims to elucidate the possible association between endotoxin (lipopolysaccharide) and zonulin (a biomarker of intestinal permeability) levels and the risk of coronary heart disease, and thus healthy aging. Serum levels of zonulin, lipopolysaccharide and soluble CD14 (a protein that binds lipopolysaccharide) were measured in disease-free centenarians, young healthy controls and patients with precocious acute myocardial infarction. Disease-free centenarians had significantly lower levels of serum zonulin (*P*<0.01) and lipopolysaccharide (*P*<0.001) than young patients with acute myocardial infarction, and had significantly lower concentrations of serum lipopolysaccharide than young healthy controls (*P*<0.05). No significant differences were found for soluble CD14 between groups. Our findings may stimulate further research into the role played by intestinal permeability and endotoxemia not only in coronary heart disease but also in lifespan modulation.

Low-grade chronic inflammation is associated with degenerative disorders including coronary heart disease (CHD), stroke and diabetes, which are among the leading causes of premature mortality in most regions of the world [1]. One possible cause of chronic inflammation is endotoxemia, a condition arising from absorption into circulation of the endotoxin lipopolysaccharide (LPS) [2], an active component of the cell wall of Gram-negative bacteria originating from the microbiota of the oral cavity and gut [2, 3] or from processed foods [4].

Translocation of LPS from the intestinal lumen into circulation can occur through transcellular or paracellular pathways [3], with the latter being regulated by a protein complex containing the enterocyte-derived protein zonulin [5]. Following specific stimuli (such as enteric bacteria and the wheat protein gliadin), zonulin reversibly disassembles intercellular tight junctions leading to increased intestinal permeability [5], which in turn may cause endotoxemia [6, 7] and low-grade chronic inflammation through activation of Toll-like receptors [7]. These phenomena are implicated in the pathogenesis of the metabolic syndrome, type 2 diabetes and CHD [2, 4, 7, 8]. Accordingly, circulating zonulin and biomarkers of endotoxemia (LPS and LPS-binding protein) have been reported to be elevated in patients with CHD [6] and type 2 diabetes [7], and to be associated with insulin resistance [7, 9, 10, 11], dyslipidemia [7], inflammation [7, 9, 11], as well as with higher waist circumference, diastolic blood pressure, fasting glucose and increased risk of metabolic diseases [12]. Moreover, circulating LPS-binding protein is positively associated with atherosclerosis [13] and has been identified as an independent predictor of CHD [14] and cardiovascular mortality [15].

In light of the aforementioned evidence and of the fact that aging is potentially associated with an increased risk of insulin resistance [16], chronic inflammation [17] and CHD [18], as well as with increased levels of circulating zonulin [19] and LPS [20], we hypothesized that both a low intestinal permeability and endotoxemia may be associated with a lower risk of CHD and contribute to healthy longevity. We therefore measured serum zonulin, LPS and soluble CD14 (an acute phase protein that mediates immune responses to LPS [21]) in disease-free centenarians (“dodgers”) [22, 23] and compared the results with young healthy controls and patients with precocious acute myocardial infarction (AMI).

## MATERIALS AND METHODS

### Participants

Three groups of subjects (described in previous publications [23-27]) were analyzed: disease-free centenarians (N=79; 39 men, 40 women), non-diabetic patients aged <40 years who had experienced AMI (N=178; 101 men, 77 women), and healthy young volunteers matched to AMI patients for age and sex (N=178; 102 men, 76 women).

All subjects were Caucasian whites ascertained to be of Italian descent (Northern Italy, mainly from Lombardy and Piedmont). The centenarians were recruited mainly via general practitioners in the community and have been previously described [23, 26, 27]. The history of past and current diseases was accurately collected, checking the centenarians’ medical documentation and the current drug therapy. Accordingly, all the centenarians were free of major age-related diseases (severe cognitive impairment, clinically evident cancer, CHD, renal insufficiency, or severe physical impairment). Healthy young controls were volunteers who were considered to be in good health, with exclusion criteria being as follows: presence of CHD or cerebrovascular disease, cancer, dementia, chronic autoimmune/inflammatory disorders, renal or hepatic failure, and major psychiatric conditions. Their compliance with study criteria was confirmed based on their family history and results of routine blood chemistry, i.e., none had hypercholesterolemia or hyper-triglyceridemia. Further, none of them was obese or had hypertension, even though 36% were current smokers.

**Table 1:** Serum biochemical parameters in the three study groups.

*Biomarkers*	Young AMI patients	Healthy young controls	Centenarians	*P*-value
LPS (EU/mL)	15.4 ± 5.2	6.1 ± 7.3	4.2 ± 5.3	<0.001
Soluble CD14 (ng/mL)	1,489 ± 339	1,438 ± 312	1,341 ± 274	ns
Zonulin (pg/mL)	7.6 ± 3.1	5.2 ± 2.7	4.0 ± 2.1	<0.001

Abbreviations: AMI, acute myocardial infarction; LPS, lipopolysaccharide: ns, not significant (*P*>0.05).

### Procedures and measures

Fasting blood samples were drawn into EDTA tubes. LPS levels were measured with the QCL-1000® chromogenic Limulus amebocyte lysate endpoint assay (Lonza, Walkersville, MD, USA). Special care was taken to avoid LPS contamination of any solution or vessel. LPS content in serum was expressed as endotoxin units (EU) per mL. Serum zonulin concentration was measured by quantitative ELISA (Immundiagnostik AG, Bensheim, Germany). Serum soluble CD14 was quantified using a commercially available ELISA (R&D systems, Minneapolis, MN, USA). The minimum detectable limits for the biochemical tests were as follows: LPS, 0.1 EU/mL; zonulin, 0.1 pg/mL; and soluble CD14: 125 ng/mL. Intra- and inter-assay coefficients of variation were as follows: LPS, 4.8% and 5.8%; zonulin, 4.1% and 6.4%; and soluble CD14, 4.1% and 6.0%.

### Statistical methods

We compared zonulin and sCD14 levels between the three groups by one-way analysis of variance (ANOVA) followed by Tukey’s post hoc test, and we used the Kruskal-Wallis test (which was skewed according to the Shapiro-Wilk test) for LPS analysis. Correlations between the study variables were assessed with Spearman’s rho test. All calculations were performed using SPSS (v17.0, SPSS Inc., Chicago, IL, USA) and GraphPad Prism (v6.0, GraphPad Inc., San Diego, CA, USA).

**Table 2:** Correlation coefficients (Spearman’s ρ) between serum biomarkers in the three study groups.

		Endotoxin	Soluble CD14	Zonulin
LPS	AMI	-	ρ = -0.06, ns	ρ = 0.48, *P* <0.001
	Controls	-	ρ = 0.09, ns	ρ = 0.19, ns
	Centenarians	-	ρ = -0.12, ns	ρ = 0.64, *P* < 0.001
Soluble CD14	AMI	ρ = -0.06, ns	-	ρ = 0.12, ns
	Controls	ρ = 0.09, ns	-	ρ = 0.08, ns
	Centenarians	ρ = -0.12, ns	-	ρ = -0.01, ns
Zonulin	AMI	ρ = 0.48, *P* <0.001	r = 0.12, ns	-
	Controls	ρ = 0.19, ns	r = 0.08, ns	-
	Centenarians	ρ = 0.64, *P* < 0.001	r = -0.01, ns	-

Abbreviations: AMI, acute myocardial infarction; LPS, lipopolysaccharide: ns, not significant (*P*>0.05).

## RESULTS

As shown in Table 1, LPS levels were significantly lower in disease-free centenarians than in AMI patients (*P*<0.001, post-hoc Dunn’s test after Kruskal-Wallis test) and healthy young controls (*P*<0.05, post-hoc Dunn’s test after Kruskal-Wallis test). Zonulin levels were significantly lower in centenarians than in AMI patients (*P*<0.01), but no differences were found when centenarians were compared with healthy controls (*P*>0.05, post-hoc Tukey’s test after ANOVA). No significant differences were found between the groups in the levels of soluble CD14 (Table 1) (*P*>0.05). The aforementioned results remained essentially unchanged when analyzing men and women separately (data not shown). On the other hand, LPS levels correlated with those of zonulin within the group of centenarians (ρ=0.64, *P*<0.001) and AMI patients (ρ=0.48, *P*<0.001), but not within healthy young controls (ρ=0.19, *P*>0.05) (Table 2).

## DISCUSSION

The present study shows that disease-free centenarians have lower serum zonulin and LPS levels than young AMI patients. Moreover, the two variables correlate with each other in centenarians and AMI patients, suggesting that intestinal hyperpermeability may cause endotoxemia, which in turn could lead to inflammation and hence insulin resistance, atherosclerosis and hypercoagulation [2, 4, 7, 8, 28, 29]. Regarding soluble CD14, although its levels have been found to be higher in patients with unstable angina [30], in individuals with the metabolic syndrome (which in turn is associated with an increased CHD risk) [21], and in HIV-infected patients (in whom CD14 levels are correlated with atherosclerosis [31] and coronary artery calcification [32]), no significant intergroup differences were found in the present study. Echoing our findings, other studies have yielded similar negative results [33, 34]. Thus, although more research is needed, our data suggest that serum levels of zonulin and LPS emerge as potential novel biomarkers of exceptional longevity. Indeed, the present cohort of centenarians has been the subject of previous research identifying other potential serum biomarkers of healthy exceptional longevity and thus of successful aging, i.e., irisin [23], vitamin D [24], eicosapentaenoic acid to arachidonic acid ratio [25], beclin-1 [26], or the combination of chemerin, fetuin-A, and fibroblast growth factors 19 and 21 [27]. Our data, coupled with the fact that aging *per se* is typically associated with elevated circulating zonulin [19], endotoxemia [20], inflammation [17], insulin resistance [16] and atherosclerosis [18], suggest that measures taken to decrease intestinal permeability and LPS translocation may help to reduce the risk of CHD and contribute to healthy lifespan. Of interest, zonulin is identical to the precursor of haptoglobin [5], an acute-phase protein that scavenges the potent oxidant free hemoglobin [5] and has anti-inflammatory activity [35]. Moreover, genetic variants of the haptoglobin gene have been associated with CHD [36] and longevity [35].

Experimental manipulation of zonulin and endotoxemia in animal models of aging and AMI, and human intervention studies are required before definite conclusions can be drawn as reverse causality cannot be firmly excluded in our study giving its observational nature. Another limitation is the lack of data on subjects’ diet and microbiota composition, since these are suspected environmental triggers of endotoxemia and zonulin expression. While we cannot yet translate our findings into clinical practice, we hope that they may inspire future studies to establish a causal link between intestinal permeability, endotoxemia and CHD.
